# Biomaterials for *Helicobacter pylori* therapy: therapeutic potential and future perspectives

**DOI:** 10.1080/19490976.2022.2120747

**Published:** 2022-09-07

**Authors:** Yongkang Lai, Wei Wei, Yiqi Du, Jie Gao, Zhaoshen Li

**Affiliations:** a Department of Gastroenterology, Shanghai Changhai Hospital, Naval Medical University, Shanghai, China; b Department of Gastroenterology, Ganzhou People’s Hospital Affiliated to Nanchang University, Ganzhou, China; c Changhai Clinical Research Unit, Shanghai Changhai Hospital, Naval Medical University, Shanghai, China

**Keywords:** Helicobacter pylori, biomaterials, nanoparticles, microspheres, hydrogels

## Abstract

*Helicobacter pylori* (*H. pylori*) is the main cause of gastric adenocarcinoma. However, the traditional antibiotic treatment of *H. pylori* is limited due to increased antibiotic resistance and low efficacy; low drug delivery efficiency and difficulties in eradicating *H. pylori* that is present intracellularly or in biofilms cause further setbacks. Biomaterials that can protect drugs against stomach acid, target lesions, control drug release, destroy biofilms, and exhibit unique antibacterial mechanisms and excellent biocompatibility have emerged as attractive tools for *H. pylori* eradication, particularly for drug-resistant strains. Herein, we review the virulence mechanisms, current drug treatments, and antibiotic resistance of *H. pylori* strains. Furthermore, recent advances in the development of biomaterials, including nanoparticles (such as lipid-based nanoparticles, polymeric nanoparticles, and inorganic nanoparticles), microspheres, and hydrogels, for effective and precise therapy of *H. pylori* and different types of therapeutic mechanisms, as well as future perspectives, have also been summarized.

## Introduction

*Helicobacter pylori* (*H. pylori*), a gram-negative spiral-shaped bacterium that colonizes the gastric mucus and gastric epithelium, has been estimated to infect nearly 4.4 billion people worldwide.^1^ It was reported that *H. pylori* is usually acquired before the age of 10, and by inducing inflammation of the host in the gastric epithelium, *H. pylori* can cause a series of gastric diseases, including peptic ulcer disease, gastric adenocarcinoma, atrophic gastritis, and mucosa-associated lymphoid tissue lymphoma.^2–6^ Additionally, *H. pylori* is closely associated with iron deficiency anemia, idiopathic thrombocytopenic purpura, and vitamin B12 deficiency.^7–9^ In 2015, the Kyoto Global Consensus Report formally defined *H. pylori* as an infectious disease and recommended treatment for all patients with it because it plays an important role in the development of gastric adenocarcinoma.^10^ In addition, a nationwide multicenter study has also indicated *H. pylori* infection to be a high-risk factor for gastric cancer.^6^ An updated meta-analysis of randomized controlled trials indicated that its eradication could reduce the incidence and mortality of gastric cancer by 46% and 39%, respectively.^11^ Furthermore, recent guidelines recommend the treatment of individuals infected with *H. pylori* to prevent gastric cancer.^2,3,12^ Therefore, the eradication of *H. pylori* from the global population is of great significance.

Currently, regimens for *H. pylori* eradication are based mainly on pharmacological treatments that include antibiotics.^3^ Early experiments have indicated a high susceptibility of *H. pylori* to antibiotics in vitro; however, it is difficult to achieve similar effects in vivo.^13^ This could be attributed to the acidic environment of the stomach (which weakens the bactericidal effects of antibiotics) and the thick gastric mucosa (which makes it difficult for most antibiotics to reach the target effectively).^13^ Therefore, treatments to efficiently eradicate *H. pylori*, such as clarithromycin triple therapy and bismuth quadruple therapy, involve the simultaneous administration of several drugs for long periods (usually 7–14 days).^3^ However, complex drug eradication programs are associated with several limitations, including large pill burdens, adverse effects, and high cost,^14^ which cause reduced adherence and low cure rates. Additionally, the progressive development of antimicrobial resistance due to the widespread misuse of antibiotics seriously undermines treatment efficacy.^14,15^ Moreover, persistent and slow-growing infections enable *H. pylori* to form biofilms or survive inside epithelial cells and macrophages by regulating autophagy, making their eradication by antibiotics extremely challenging.^16^ Therefore, new treatment strategies that can overcome the acidic gastric environment, increase drug delivery efficiency, and eradicate *H. pylori* that exists intracellularly or in biofilms are urgently needed for effective alternatives.

Biomaterials formed by synthetic material engineering exhibit several important applications in cancer treatment, antibacterial, immunomodulatory, wound healing processes, and so on.^17–20^ Biomaterials have also been used for the treatment of *H. pylori* infection due to their unique properties compared to conventional antibiotic therapy. First, biomaterials can conjugate with or encapsulate conventional antibiotics, which can reduce the administered dose and side effects and enhance bioavailability. In addition, the site-specific targeting of *H. pylori* and controlled drug release by biomaterials also improve antibacterial efficacy and slow down the emergence of drug-resistant bacteria.^21^ Additionally, biomaterials (such as inorganic nanoparticles) can enter bacterial cells directly and destroy biofilms, eliminating the *H. pylori* present there.^22,23^ Some biomaterials (such as linolenic acid and metal ions) exhibit unique antibacterial mechanisms (they affect the metabolic activities of *H. pylori*, change its membrane permeability or produce reactive oxygen species (ROS)) and can eradicate *H. pylori* without any loaded drugs; they hinder the development of drug resistance in *H. pylori*.^24–26^ Currently, new *H. pylori* treatment strategies, including Chinese herbal medicines, probiotics, antimicrobial peptides, and urease inhibitors, are also being studied by researchers to eradicate *H. pylori*. However, compared with biomaterials, these strategies are either cytotoxic, intolerant to gastric acid and pepsin, or have a lower eradication rate.^27–30^ Therefore, biomaterials are effective alternatives for efficient *H. pylori* therapy; liposomes, nanostructured lipid carriers, nanoemulsions, polymeric nanoparticles, inorganic nanoparticles, microspheres, and hydrogels are commonly used.^31–33^

Herein, the pathogenic mechanisms of *H. pylori* infection have been described, along with its current drug treatments and drug-resistance mechanisms. Furthermore, recent progress in biomaterials for *H. pylori* eradication has been summarized. Thus, biomaterials can be used for the effective and precise therapy of *H. pylori* infections in the future.

### H. pylori

#### H. pylori and its virulence mechanisms

Isolated and cultured by Warren and Marshall for the first time, *H. pylori* is a spiral microaerobic bacterium with five to seven flagella and demanding growth conditions.^34^ *H. pylori* infection will first cause chronic gastritis and then gradually lead to gastric ulcers and gastric atrophy;^35^ in severe cases, it can lead to gastric cancer.^36^ It has been reported that there is a parallel relationship between H. pylori and gastric cancer mortality, and eradication of H. pylori is closely related to the prevention of gastric cancer.^6,37,38^

**Figure 1.**
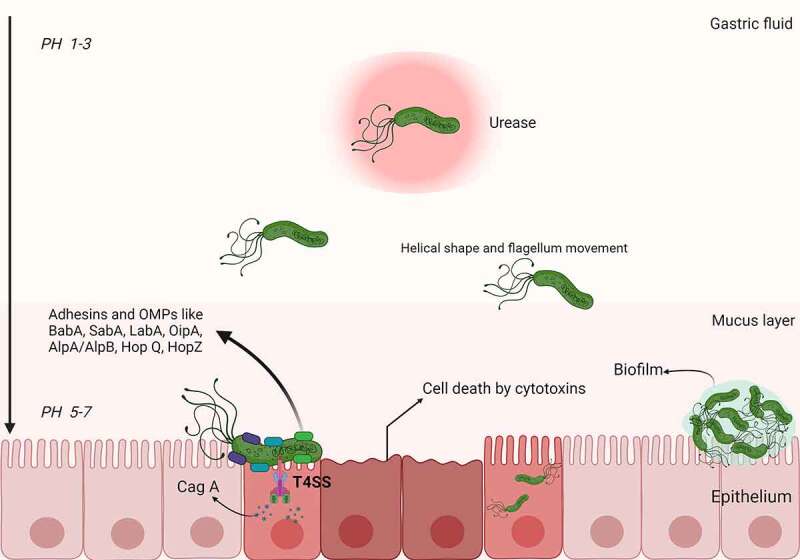
**Schematic representation of *H. pylori* pathogenesis and virulence factors**. Upon entering the stomach, *H. pylori* begins to produce urease, which promotes the hydrolysis of urea to synthesize gastric acid. In this way, bacteria can resist gastric acidity and survive in the stomach. With the help of its helical shape and flagellum movement, *H. pylori* can easily penetrate the mucus layer and reach the gastric epithelium. After reaching the gastric epithelium, *H. pylori* colonizes gastric epithelial cells under the synergistic action of a variety of adhesins and outer membrane proteins (e.g., BabA, SabA, LabA, OipA, AlpA/AlpB, HopQ, and HopZ). Toxins (e.g., Cag A) are then injected into the host cells through the T4SS, leading to an inflammatory response. In addition, *H. pylori* can form biofilms composed of bacteria and a self-secreted extracellular matrix that adheres to inert and living surfaces; growth in biofilms facilitates the development of antibiotic resistance in *H. pylori.*

The pathogenesis of *H. pylori* infection is shown in Figure 1. *H. pylori* produces urease upon entering the stomach, which promotes the hydrolysis of urea, forming ammonia and carbon dioxide. Ammonia neutralizes gastric acid, while carbon dioxide helps bacteria survive in the stomach by resisting gastric acidity. In this way, the bacteria can resist gastric acidity and survive in the stomach. *H. pylori*, with the help of its helical shape and flagellar movement, easily penetrates the mucus layer to reach the gastric epithelium,^39^ where it colonizes gastric epithelial cells under the synergistic action of adhesins and outer membrane proteins (such as BabA, SabA, LabA, OipA, AlpA/AlpB, HopQ, HopZ).^40–42^ Then, the proteins and toxins located in the host cells subsequently cause an inflammatory response.^43^

Among the many pathogenic factors of *H. pylori*, cytotoxin-associated gene A (*cagA*) and vacuolating cytotoxin A (VacA) are the most extensively investigated virulence factors. Cytotoxin-associated protein A (CagA) is a protein produced by *H. pylori*, with masses in the range of 120–145 kDa. CagA, along with the type 4 secretion system (T4SS), is encoded by the cag pathogenicity island (*cagPAI*), which comprises approximately 40 kb of chromosomal DNA.^44^ According to the presence and absence of CagA, *H. pylori* can be classified into two subtypes, CagA-positive and CagA-negative. After attachment to gastric cells, the CagA-positive *H. pylori* injects CagA into the host cells through the T4SS, similar to a needle. Compared to those infected with CagA-negative *H. pylori* strains, patients with a CagA-positive *H. pylori* infection have a higher risk of gastric cancer or peptic ulcer disease.^45^ Xie et al. revealed autophagy was decreased in the duration of H. *pylori* infection in a CagA-dependent manner, resulted in ubiquitination degradation of RAD51 and genomic instability in gastric cells, which inducing progression of gastric epithelial cells to cancer.^45^ VacA is a cytotoxin encoded by the *vagA* gene; its main function is cell vacuolation through pores on the epithelial cell membrane. Additionally, it helps *H. pylori* resist immune clearance, improves its viability, and enables the host to continue infecting through membrane depolarization, apoptosis stimulation, intercellular adhesion, and T-cell obstruction.^40^ VacA also interferes with autophagy, enabling the survival of *H. pylori* in host cells.^46^

## Current drug treatments and antibiotic resistance of H. pylori

The current regimens for *H. pylori* treatment integrate one PPI with a combination of antimicrobial agents (Table 1). According to the Maastricht V/Florence consensus report, in areas of low (<15%) clarithromycin resistance, the standard PPI-clarithromycin-containing regimen is recommended as the first-line treatment, whereas the bismuth-quadruple regimen is recommended in areas of high (>15%) clarithromycin resistance.^3^ After failure of the first-line treatment (clarithromycin-containing triple therapy or bismuth quadruple therapy), fluoroquinolone-containing (for example, levofloxacin) triple or quadruple therapy is recommended as the second-line treatment. After failure, the patient is recommended to undergo susceptibility testing or molecular determination to guide further treatment.^3^ In addition to conventional regimens, researchers have modified drug-administration sequences and created sequential, hybrid, and concomitant therapies. However, due to resistance to clarithromycin and metronidazole, sequential therapy gradually fades out of the field of research.^12,49^ Although hybrid and concomitant therapies exhibit satisfactory *H. pylori* eradication rates,^50,51^ they involve complex drug administration, which may affect patient compliance, and the exposure of patients to at least one unnecessary antibiotic, which may promote *H. pylori* resistance. Thus, the promotion and application of these regimens should be investigated further.^49,52^ Graham et al. reported a rifabutin-based triple therapy randomized controlled study and indicated that the regimen is unaffected by clarithromycin or metronidazole resistance.^53^ However, this study excludes individuals of Asian descent; therefore, the applicability of rifabutin-based triple therapy to Asian people, who have a high risk of stomach cancer, remains unknown.^53^ High-dose PPI-amoxicillin dual therapy and vonoprazan (a new potent acid-inhibitory drug) triple or dual therapy are popular new treatment methods. Numerous studies have confirmed the high *H. pylori* eradication rates and low side effects of both methods.^48,54–56^ Interestingly, Horii et al. reported that vonoprazan dual therapy has a low impact on gut microbiota.^57^ However, vonoprazan is expensive; thus, economic aspects should be considered when using it for treatment. Additionally, probiotics have been poven to improve the eradication effect.^58^ A study recruiting 234 *H. pylori*-positive gastritis patients from seven local centers indicated that probiotic administration before or after standard triple therapy improves *H. pylori* eradication rates.^58^ In addition, to reduce the chance of reinfection after *H. pylori* eradication, the strategy of “family-based *H. pylori* management” could be vital.^59^

**Table 1.** The current regimens for *H. pylori* treatment.

Therapy	Components (usual Doses and Frequencies)	Days (n)	Reference
Clarithromycin triple therapy	Clarithromycin (500 mg bid)+amoxicillin (1 g bid) or metronidazole (500 mg bid)+PPI (standard dose bid)	14	^47^
Bismuth quadruple	Bismuth (300 mg qid)+metronidazole (500 mg tid or qid)+tetracycline (500 mg qid)+PPI (standard dose bid)	10 or 14	^47^
Levofloxacin triple	Levofloxacin (500 mg qd)+ amoxicillin (1 g bid)+ PPI (standard dose bid)	14	^12^
Levofloxacin quad	Levofloxacin (500 mg qd)+ PPI (standard dose bid)+ 2 antibiotics (multiple variations exist)	10 or 14	^2^
Concomitant therapy	Clarithromycin (500 mg bid)+ amoxicillin (1 g bid)+metronidazole or tinidazole (500 mg bid)+PPI (standard dose bid)	14	^12^
Sequential therapy	PPI (high-dose* qd) + amoxicillin (1 g bid); then PPI (standard dose bid)+ clarithromycin (500 mg bid)+nitroimidazole (500 mg bid)	5–7 then 5–7	^12^
Rifabutin triple	Rifabutin (150 or 300 mg qd), amoxicillin (1 g bid), PPI (standard dose bid)	10 or 14	^47^
High-dose dual	Amoxicillin (2 − 3 g daily in 3–4 split doses) PPI (high-dose* bid)	14	^47^
Vonoprazan dual therapy	Vonoprazan (20 mg bid)+amoxicillin (750 mg bid)	7	^48^
Vonoprazan triple therapy	Vonoprazan (20 mg bid) + amoxicillin (750 mg bid) + clarithromycin (200 mg bid)	7	^48^

*PPI standard dose is as follows: pantoprazole 40 mg, esomeprazole 20 mg, omeprazole 20 mg, lansoprazole 30 mg, rabeprazole 20 mg.dexlansoprazole 30 mg, “High-dose” implies double the standard dose; qd: once daily; bid: twice daily; tid: thirce daily; qid: four times daily

**Figure 2.**
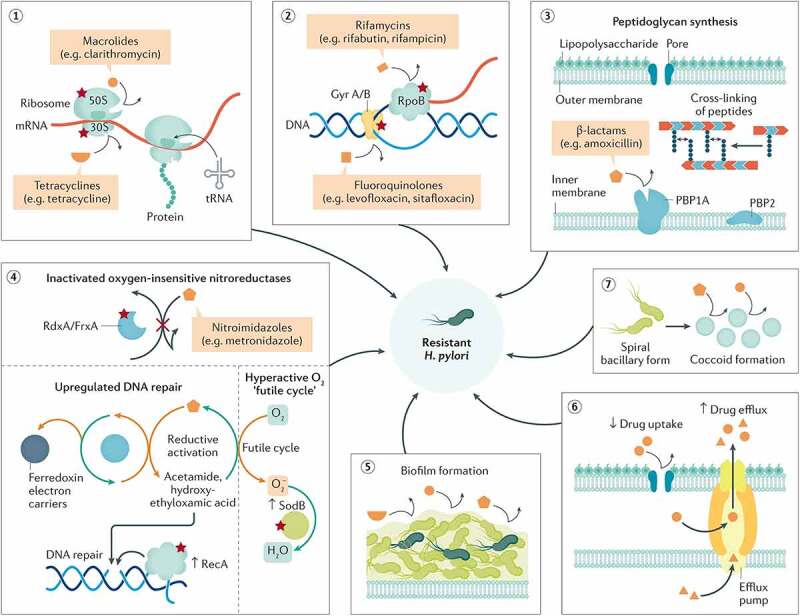
**Biological attributes of resistance in *H. pylori***. The figure shows the biological attributes that drive antibiotic resistance in the *H. pylori* species (red stars denote the possibility of resistance mutation). It comprises mainly structural changes in genetic sequences that disrupt the cellular activity of antibiotics by altering the drug target (1–3), inhibiting drug activation within the cells (4), biofilm formation (consisting of a matrix of polysaccharides that constitute a multifactorial barrier to drug penetration and activity) (5), reduced drug uptake and increased drug efflux (6), or coccoid formation that generates ultrastructural and metabolic changes that prevent cellular penetration and activity of antibiotic molecules (7). All these attributes are not mutually exclusive but possibly coexist in different strains, jointly leading to three patterns of resistance: single drug resistance (SDR), multidrug resistance (MDR), and heteroresistance (HR). **Reproduced with permission from ref**,^15^ **© Tshibangu-Kabamba E (2021).**

However, rapidly increasing antibiotic resistance has reduced the efficacy of traditional antibiotic therapy against *H. pylori*.^60^ At present, the drug resistance of *H. pylori* mainly refers to resistance against antibiotics used in eradication therapies (such as clarithromycin, fluoroquinolones, metronidazole, amoxicillin, tetracycline, and rifabutin). The mechanism of antibiotic resistance primarily involves structural changes in the gene sequence, the destruction of antibiotic activity by changing drug targets, and the inhibition of intracellular drug activation, affecting drug-efflux mechanisms and causing enzymatic deactivation of the drug (Figure 2).^15^ In addition, the acidic environment of the stomach and the presence of thick gastric mucosa (approximately 200 μm) also affect the efficacy of antibiotic delivery, which reduces the cure rate.^15^ Furthermore, *H. pylori* can form biofilms composed of bacteria and a self-secreted extracellular matrix that adhere to inert or living surfaces.^61^ Biofilms play an imperative role in antibiotic resistance by reducing drug penetration, promoting gene mutation, and overexpressing efflux pumps involved in drug resistance.^15^ The matrix includes an effective and nonspecific barrier, which prevents drug penetration and promotes the overexpression of efflux pumps in the process of drug resistance; thus, encapsulating *H. pylori* can significantly enhance its viability. Compared to free-floating strains, *H. pylori* growth in biofilms may occur for a longer time, with a greater susceptibility to developing antibiotic resistance.^62,63^ Given the aforementioned reasons, investigating alternatives to increase drug-delivery efficiency and eradicate *H. pylori* present intracellularly or in biofilms may be the key to resolving several issues related to its rapidly increasing drug resistance.

## Biomaterials

Biomaterials possess many unique properties, such as a large specific surface area, targeted lesion sites, satisfactory biocompatibility, and excellent stability;^64^ their application in disease treatment is an important aspect of modern medicine. Owing to their unique antibacterial mechanisms and efficiency in eradicating drug-resistant strains, biomaterials are gradually being used in *H. pylori* treatment. Currently, biomaterials are mainly used as delivery systems for drugs to eradicate *H. pylori*, which can increase the drug delivery efficiency. Apart from encapsulating antibacterial agents, biomaterials (such as lipid nanoparticles, chitosan nanoparticles, and inorganic nanoparticles) have been directly applied in *H. pylori* treatments because of their inherent antibacterial activity. Herein, recent studies on the progress of biomaterials in the treatment of *H. pylori* infection have been described and summarized.

## Nanoparticles

Nanoparticles, defined as nanosized delivery systems that encapsulate drugs, can increase drug solubility and modulate drug-release characteristics and are valuable tools in fighting antibiotic resistance.^21^ Based on the matrix material, nanoparticles have been classified into several categories, such as lipid-based nanoparticles (including liposomes, nanostructured lipid carriers, and nanoemulsions), polymeric nanoparticles, and inorganic nanoparticles.

### Lipid-based nanoparticles

Lipid-based nanoparticles (including liposomes, solid lipid nanoparticles, nanostructured lipid carriers, and nanoemulsions) are among the most widely used drug delivery systems for antimicrobial therapy.^65,66^ With compositional similarity to cell membranes, lipid-based nanoparticles exhibit high biocompatibility and low toxicity.^67,68^ Combined with other materials, they can present excellent features, such as target sites and controlled drug release, enabling a high drug-delivery efficiency and low occurrence of antibiotic resistance in *H. pylori*.^69^ Notably, lipid-based nanoparticles (such as liposomes) can enter cells and kill *H. pylori* inside epithelial cells, macrophages, and biofilms.^70^ In this section, diverse lipid-based nanoparticles (mainly including liposomes and nanostructured lipid carriers) used for the eradication of *H. pylori* have been outlined, focusing on their stability, cargo transportation, and antimicrobial activity.

### Liposomes

**Figure 3.**
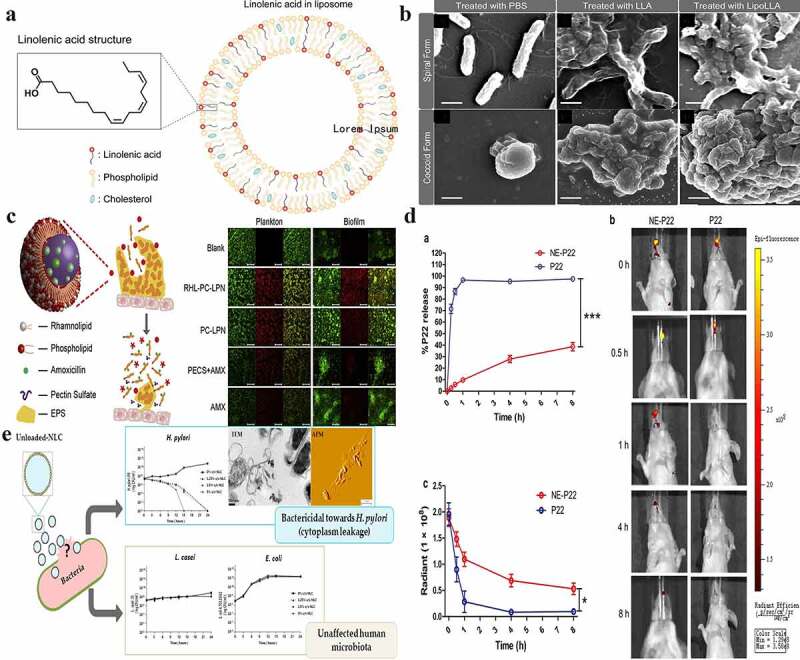
**Lipid-based nanoparticles used for *H. pylori* therapy. a)** The schematic drawing shows the molecular structure of LipoLLA, which is composed of phospholipids, cholesterol, and LLA. **b)** Morphologies of *H. pylori* SS1 bacteria in the spiral and coccoid forms exposed to different treatments. **c)** The strategy for lipid polymer nanoparticles to eradicate bacterial biofilms and the eradication of *H. pylori* plankton in biofilms by nanoparticles. Blank: the blank group with only bacteria; RHL-PC-LPN: amoxicillin-loaded lipid polymer nanoparticles; LPN: using rhamnolipid and phospholipids as mixed lipids; PC-LPN: amoxicillin-loaded LPN: only using phospholipids as the lipid component; PECS + AMX: a mixture of sulfated pectin and amoxicillin; AMX: amoxicillin only; **d)** The in vitro and in vivo release of NE-P22; **a**. In vitro release profile of NE-P22; **b**. In vivo fluorescence imaging of FITC-labeled P22 in mice; **c**. Quantification of fluorescence intensity; **e)** Nanostructured lipid carriers kill *H. pylori* by changing cell-membrane permeability at low concentrations, even without any loaded drug; the eradication does not affect other gut microbiota (such as L. casei and E. coli). (a) **and** (b) **Reproduced with permission from ref**,^24^ **© 2012, American Chemical Society;** (c) **Reproduced with permission from ref**,^78^ **© 2015 Elsevier B.V**. (d) **Reproduced with permission from ref**,^79^ **© 2019, The Author**(s); (e) **Reproduced with permission from ref**,^80^ **© 2018 Elsevier B.V.**

Liposomes are defined as smooth, continuous, and bilayered structures that contain mainly phospholipid molecules.^71^ As delivery systems, they exhibit numerous advantageous properties. First, they are composed of phospholipid bilayers encapsulating water-phase vesicles, and liposomes have high elasticity and good biocompatibility. Additionally, they exhibit high drug-loading and entrapment capacity as the water-soluble and fat-soluble drugs are dissolved in the water phase and lipid membrane, respectively. Furthermore, phospholipids themselves are cell-membrane components; thus, liposomes are nontoxic with high bioavailability.^21,72,73^ As one of the most widely investigated antibiotic delivery systems, liposomes are often used to deliver antibiotics (such as amoxicillin, furazolidone, doxycycline, and metronidazole) in *H. pylori* treatments.^74–77^ In particular, liposomes are ideal vehicles for delivering antimicrobial lipids (such as lauric acid, myristoleic acid, linoleic acid, and linolenic acid (LLA)), making it difficult for *H. pylori* to develop resistance. For example, Obonyo et al. designed a system named liposomal linolenic acid (LipoLLA) by incorporating lauric acid, myristoleic acid, linoleic acid, and LLA, which solves the problem of poor solubility of LLA in aqueous solutions (Figure 3a).^24^ After incubation with LipoLLA at a concentration of 67 μg/mL for 30 min, 99.9% of *H. pylori* were killed. Notably, LipoLLA can also kill the dormant form (the coccoid form) of *H. pylori* by damaging its membrane (Figure 3b).^81^ Additionally, *H. pylori* treated with LipoLLA did not acquire drug resistance during the 10-day period, while drug resistance to LLA was established on day 3.^24^ Apart from delivering anti-*H. pylori* drugs alone, liposomes have also been integrated with other materials (such as pectin and monoclonal antibodies against *H. pylori*) to exhibit target specificity and antiadhesion.^76,82^ For example, Gottesmann et al. reported the fabrication of pectin-coated liposomes with encapsulated amoxicillin that utilize a specific interaction of pectin with mucins (BabA) and the surface structures of *H. pylori*.^76^ Their results showed that pectin-coated liposomes exhibit stronger bactericidal effects than nonpectin-coated liposomes.^76^

However, there are some challenges associated with the use of liposomes to eradicate *H. pylori*. First, liposomes alone exhibit low encapsulation efficiencies for antibiotics,^83,84^ which may be because liposomes are too unstable for gastric antibiotic delivery.^32^ Adding cholesterol to liposomal formulations can improve liposome stability and increase the interactions between liposomes and *H. pylori*.^76^ However, excessive cholesterol may disrupt the linear structure of liposomal membranes, decreasing the encapsulation efficiency and drug release rate.^74^ Thus, selecting an appropriate proportion of cholesterol is vital for generating stable liposomes. Alam *et al*. investigated the effects of different cholesterol amounts on the encapsulation efficiency and drug release rate of different liposome formulations. The formulation containing 5 mg of furazolidone, 53 mg of cholesterol, and 106 mg of lipid exhibits optimal encapsulation and drug release efficacy.^74^ Combining liposomes with polymers is another helpful way to improve their stability. For example, Cai et al. reported amoxicillin-loaded liposome-polymer hybrid nanoparticles with high stability in the acidic environment of the stomach.^78^ Interestingly, the addition of rhamnolipids imparted a strong biofilm-disrupting capacity to the nanoparticles (Figure 3c).^78^ Second, the complex manufacturing processes of liposomes increase their cost and lower their production, limiting their use in routine clinical applications.^85^ Hence, it is important to develop a facile and economical liposome manufacturing process.

In summary, the anti-*H. pylori* mechanisms of liposomes exhibit the following features. First, they can protect drugs from stomach acid degradation and enhance their solubility. Second, they can increase membrane permeability and alter the membrane structure of *H. pylori*. However, the use of liposomes is limited by their complex preparation processes.

### Nanoemulsions

Nanoemulsions are kinetically stable colloidal-particulate delivery systems formed by the dispersion of two immiscible liquids (such as oil and water) that are stabilized by surfactants and typically exhibit sizes in the range of 20–200 nm.^66^ Generally, nanoemulsions are classified into three types: oil-in-water, water-in-oil, and bicontinuous.^86^ Unlike liposomes, nanoemulsions not only exhibit high biocompatibility but also present long-term stability and are fabricated via simple processes (unlike liposomes), enabling their large-scale production and long-term preservation.^66^ In regard to antimicrobials, nanoemulsions exhibit antibacterial activity and can destroy bacteria without the addition of any antimicrobial substances.^86^ Furthermore, they improve the stability, solubility, and bioavailability of loaded (especially lipophilic) drugs.^86,87^ For instance, Tran et al. developed a nanoemulsion-based delivery system loaded with erythromycin, which exhibits low solubility in water and is easily destroyed in acidic environments, for the eradication of *H. pylori*. Their results showed that the stability of erythromycin was significantly enhanced by the nanoemulsion system.^87^ Additionally, nanoemulsions exhibit effective adjuvant activity for vaccines. Yang et al. designed an intranasal HpaA epitope peptide-loaded nanoemulsion delivery system to protect against *H. pylori* infection.^79^ They found that this system could extend antigen release and induce Th1 responses, which indicated that nanoemulsions are an ideal delivery system for intranasal vaccines against *H. pylori* (Figure 3d).^79^ However, although nanoemulsions are widely used for combating bacterial growth and are easy to produce/preserve,^86^ there are very few studies on the eradication of *H. pylori* using them. Therefore, the applicability of nanoemulsions as effective alternatives for *H. pylori* therapy requires further investigation.

### Nanostructured lipid carriers

Nanostructured lipid carriers are the second generation of solid lipid nanoparticles produced from solid and liquid lipids.^68^ They can be grouped into three classes (imperfect, amorphous, and multiple oil-in-solid fat-in-water) according to their mixture composition and fabrication method.^88^ Nanostructured lipid carriers exhibit good biocompatibility and stability and can lead to high storage capacities and encapsulation efficiencies.^21,88^ For example, Seabra et al. fabricated docosahexaenoic acid (DHA)-loaded nanostructured lipid carriers that inhibit *H. pylori* growth at a very low concentration (25 mM), indicating that the nanoparticles effectively protect the properties of DHA. In addition, they also found that the antibacterial mechanism of DHA against *H. pylori* primarily involved the destruction of the bacterial cell membrane.^89^ Interestingly, in a later study by Seabra et al., they found that nanostructured lipid carriers without any loaded drugs can eradicate *H. pylori*, even at very low concentrations.^80^ To explore the underlying mechanism, *H. pylori* was cultured with nanostructured lipid carriers (1.25%) and analyzed by transmission electron microscopy and atomic force microscopy, and they found that most bacterial membranes were disrupted after 12 h (Figure 3e). This indicated that nanostructured lipid carriers can kill *H. pylori* by destroying their membranes. Furthermore, nanostructured lipid carriers have been demonstrated to have the ability to be specific to *H. pylori* and do not affect gut microbiota.^80^

In summary, lipid-based nanoparticles exhibit high biocompatibility and can enhance the solubility and bioavailability of anti-*H. pylori* drugs. Additionally, they can kill bacteria inside cells or biofilms via endocytosis or fusion with the cell membrane. However, lipid-based nanoparticles exhibit drug leakage (which affects the control of drug release)^90^ and involve complicated preparation processes (e.g., liposomes), making routine clinical applications challenging. Therefore, developing methods to improve their controlled drug-release capacity and simplifying their synthetic procedures could make lipid-based nanoparticles an effective alternative for *H. pylori* therapy in the future.

### Polymeric nanoparticles

**Figure 4.**
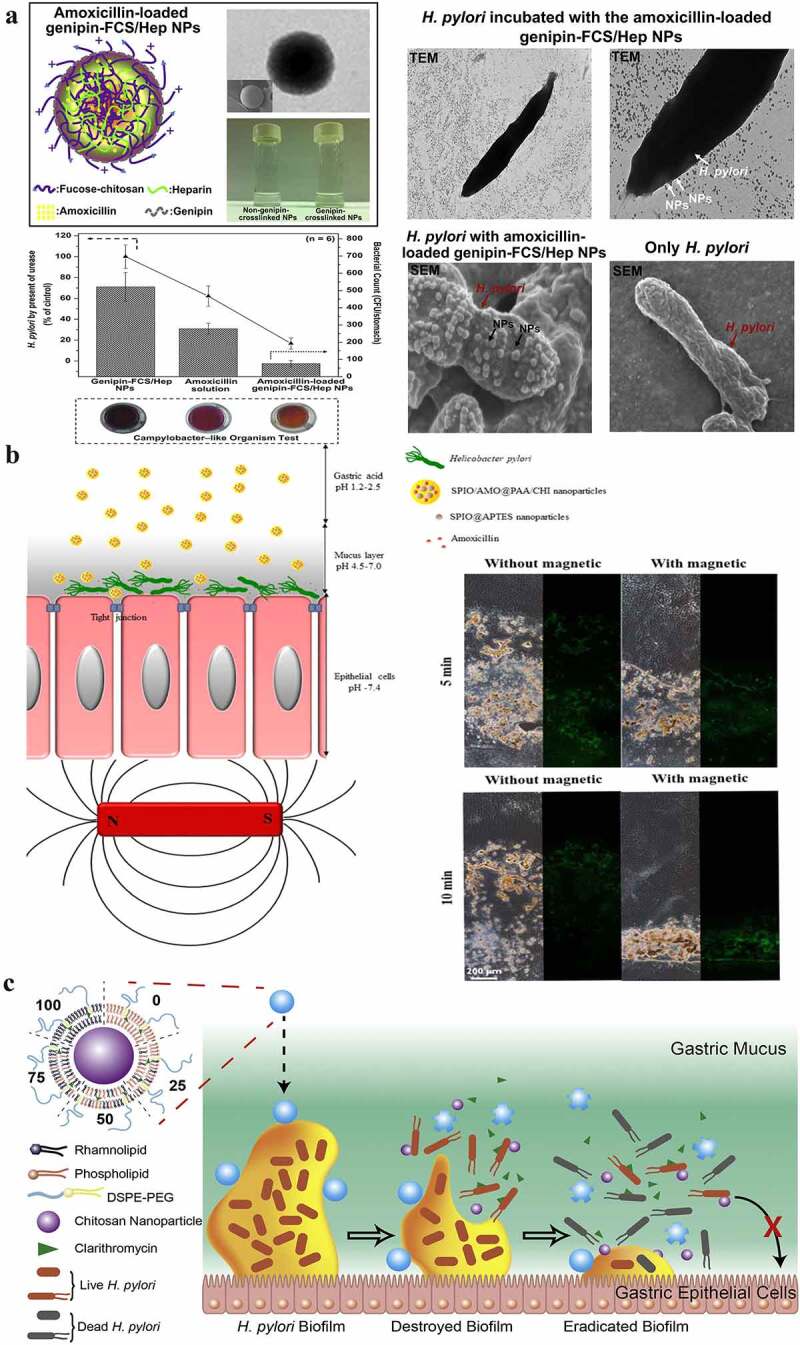
**a)** A representation of amoxicillin-loaded genipin-FCS/Hep NPs, the strategy and observations for *H. pylori* eradication using them, and the effects of amoxicillin solution alone and with/without amoxicillin-loaded genipin-FCS/Hep NPs in an *H. pylori*-induced gastric infection mouse model; **b)** Schematic illustration of SPIO/AMO@PAA/CHI nanoparticles in the gastric acid of gastric lumen; they adhere to and penetrate the mucus layer, become unstable, and release amoxicillin at the site of *H. pylori* infection. The distribution of the prepared FITC-SPIO/AMO@PAA/CHI in the mucin layer with/without exposure to a magnetic field for 5 and 10 min. **c)** Schematic illustration of the structure and bacterial biofilm eradication process of lipid polymer nanoparticles. (a) **Reproduced with permission from ref**,^96^ **© 2013, Elsevier Ltd**. (b) **Reproduced with permission from ref**,^95^ **© 2020, American Chemical Society** (c) **Reproduced with permission from ref**,^98^ **© 2019, Elsevier B.V.**

Polymeric nanoparticles are produced using polymeric materials (such as chitosan, ethylcellulose, and poly(lactic-co-glycolic acid)) and colloidal organic compounds in nanosizes.^91,92^ Compared with lipid-based nanoparticles, polymeric nanoparticles exhibit higher loading capacities and mechanical stabilities.^93^ Chitosan, the most widely studied natural cationic polysaccharide polymer, exhibits good adhesion and antibacterial properties.^94^ Interestingly, chitosan nanoparticles can bind to *H. pylori* through electrostatic interactions and inhibit its growth by changing the permeability of bacterial cell membranes, even without any drug loading. For example, Luo et al. have developed chitosan nanoparticles that exhibit the highest bacteriostatic activity at pH 4. Notably, they found that chitosan nanoparticles with a 95% degree of deacetylation exhibited a higher anti-*H. pylori* effect than those with an 88.5% degree of deacetylation, indicating that a stronger antibacterial effect is exhibited by chitosans with a higher degree of deacetylation.^94^ Furthermore, chitosan and its derivatives can be utilized as drug delivery systems because of their special mucoadhesive properties (due to the electrostatic interaction between the positively charged chitosan and negatively charged cells/mucus), which can significantly extend the residence time of drugs in the stomach.^95,96^ For example, Lin et al. developed pH-responsive chitosan nanoparticles by integrating chitosan and heparin.^97^ Their results showed that the nanoparticles enhanced the drug-membrane epithelium interaction through the electrostatic interaction between chitosan and mucosa and promoted gastric ulcer healing by the effect of heparin.^97^ Under acidic conditions (pH in the range of 1.2–6.5), heparin and chitosan form spherical polyelectrolyte complexes that disintegrate upon reaching the infection site (where the pH is approximately neutral).^97^ In their later study, they fabricated amoxicillin-loaded genipin-cross-linked fucose-conjugated chitosan/heparin nanoparticles (genipin-FCS/Hep nanoparticles).^96^ Fucose can directly adhere to *H. pylori*, binding to it, while genipin can slow down the release of amoxicillin. The results showed that the effect of amoxicillin-loaded genipin-FCS/Hep nanoparticles in clearing *H. pylori* infection was stronger than that of amoxicillin alone (Figure 4a).^32,96^ Targeting lesions directly is an effective strategy to increase drug delivery efficiency and reduce the occurrence of antibiotic resistance in *H. pylori*. Based on a similar idea, Lin et al. fabricated fucose-chitosan/heparin nanoparticles to deliver berberine, a traditional Chinese medicine with antibacterial and anti-infective properties.^99^ Their results demonstrated that targeting *H. pylori* directly enables more significant *H. pylori* growth inhibition than other untargeted strategies.^99^ In addition, chitosan has been combined with other materials to form systems that exhibit unique characteristics with high antibacterial activity. For example, Yang et al. developed superparamagnetic iron oxide nanoparticles coloaded with amoxicillin and chitosan/polyacrylic acid particles.^95^ Polyacrylic acid particles can compete with amoxicillin to bind to chitosan, enabling the continuous release of amoxicillin in the stomach. What is interesting in this study is the role of superparamagnetic iron oxide in this system, which increased the ability of nanoparticles to penetrate the H. pylori membrane and prolonged the residence time of nanoparticles in the stomach after exposure to the magnetic field (Figure 4b).^95^ Rhamnolipid-coated chitosan hybrid nanoparticles are advantageous because they integrate the high drug-loading efficiency and stability of chitosan nanoparticles with the biofilm-destruction ability of rhamnolipid layers. For example, Li et al. and Arif et al. have established rhamnolipid-chitosan hybrid nanoparticles loaded with either clarithromycin or amoxicillin and applied them to gastric delivery; both studies indicate a strong ability (89% and 99%, respectively) to eradicate *H. pylori* biofilms under the mucus layer at minimal inhibitory concentrations of 32 and 132 µg/ml, respectively (Figure 4c)^98,100^ This further confirms the versatility of biomaterials as anti-*H. pylori* agents; they exhibit different properties in combination with different materials. In addition to chitosan, other polymers (such as ethyl cellulose) have also been studied.^101^

Polymers are also used as delivery vehicles for mRNA, DNA, and/or adjuvants in vaccines in various forms and morphologies because of their excellent stability.^102^ For example, Chehelgerdi et al. prepared a cagW‑based DNA vaccine loaded with chitosan nanoparticles;^103^ upon injecting mice with this vaccine, the expression of cagW increased on the 7th day and then gradually decreased, reaching a minimum on the 45th day. Furthermore, they found that vaccination significantly increases cellular immunity. Interestingly, during the *H. pylori* challenge, the vaccinated mice can clear the *H. pylori* infection. This suggested that the vaccine exhibited a protective effect against *H. pylori* in mice.^103^ In addition to chitosan, polymers such as HP55 (a special type of hydroxypropyl methylcellulose phthalate)/D and poly(lactic-co-glycolic acid) (PLGA) have also been used as delivery systems for vaccines.^104^ For example, Tan et al. designed an oral dual-antigen epitope and dual-adjuvant vaccine based on the urease of *H. pylori* and cholera toxin B subunits and delivered it with HP55/PLGA nanoparticles.^104^ This vaccine exhibits pH-dependent antigen release, with a release rate of less than 5% in acidic environments (pH ≤ 4.5) and 75% in neutral environments (pH = 7.5). In vivo results indicate that vaccine-encapsulated HP55/PLGA nanoparticles elicit high levels of systemic and localized antigen-specific antibodies and Th1/Th17-biased immune responses. During *H. pylori* challenge, mice injected with this vaccine exhibited 43% complete protection.^104,105^ Oral vaccines are considered to be the best way to elicit an immune response in the human stomach. However, owing to the acidic environment in the stomach, oral vaccines are easily hydrolyzed, resulting in low antigen uptake efficiency. The use of polymer nanoparticles to deliver oral vaccines is undoubtedly a good way to solve this problem.

Although efforts to develop an *H. pylori* vaccine began in the early 1990s,^106^ effective vaccines are currently lacking. The main reason for this could be their inability to induce a completely protective immune response against *H. pylori*.^107^ Moreover, the necessity for developing an *H. pylori* vaccine remains controversial. Nevertheless, rapidly increasing drug resistance rates, the high prevalence of *H. pylori* infection, and frequent reinfections due to ineffective antibiotic treatment indicate that childhood vaccination to prevent *H. pylori* infection could be an effective and economical strategy. This is especially true for countries with a high incidence of gastric cancer (such as China and Japan).^108^ At present, identifying suitable antigens and adjuvants is vital to facilitate the development of *H. pylori* vaccines. The delivery of *H. pylori* vaccines using nanotechnology could improve vaccine delivery effectiveness and prevent its degradation, thereby improving vaccine efficacy. With the declining success rate of current *H. pylori* treatment strategies, designing an effective vaccine could be a cost-effective way to control *H. pylori* epidemics.

To summarize, polymer-based polymeric nanoparticles are mainly investigated as drug delivery systems in *H. pylori* eradication regimens, particularly for hydrophobic drugs. They cause high drug stability and controlled release, leading to a high drug delivery efficiency and low *H. pylori* drug resistance. Some polymer-based polymeric nanoparticles, such as chitosan, exhibit excellent adhesion and the ability to change the permeability of *H. pylori* membranes, which enables chitosan nanoparticles to inhibit the growth of *H. pylori*, even without any drug loading.

### Inorganic nanoparticles

Inorganic nanoparticles are defined as inorganic salts (such as silver, gold, and zinc) or oxides with nanoscale sizes (1–100 nm); they exhibit small particle sizes, large specific surface areas, numerous surface-active centers, and strong catalytic abilities.^33,109^ As an effective strategy to eradicate *H. pylori*, anti-*H. pylori* mechanisms of inorganic nanoparticles mainly involve metal-ion release, ROS production, and disruption of the *H. pylori* cell membrane and biofilm.^32,110^ Owing to inorganic nanoparticles’ unique antimicrobial mechanism, it is difficult for *H. pylori* to develop resistance.

Among the inorganic nanoparticles, silver nanoparticles, which inhibit the urease activity of *H. pylori* and reduce biofilm formation, are the most representative.^22,111^ Gopalakrishnan et al. designed N-acylhomoserine lactonase-stabilized silver nanoparticles (AiiAAgnanoparticles) that inhibit biofilm formation by degrading quorum-sensing molecules and inhibit urea production in *H. pylori*.^22^ Interestingly, AiiAAgNPs exhibit no toxic side effects at effective concentrations (1–5 μM), but they exhibit cytotoxic effects against tumor cells at high concentrations (80–100 μM).^22^ Similarly, Camargo et al. also synthesized a silver ion-based complex (Ag(PhTSC∙HCl)_2_](NO_3_)∙H_2_O) and transported it using polymeric nanoparticles.^111^ Their results showed that these complexes exhibited high efficacy against floating *H. pylori* and *H. pylori* present in biofilms, with no in vivo toxicity for Galleria mellonella.^111^

**Figure 5.**
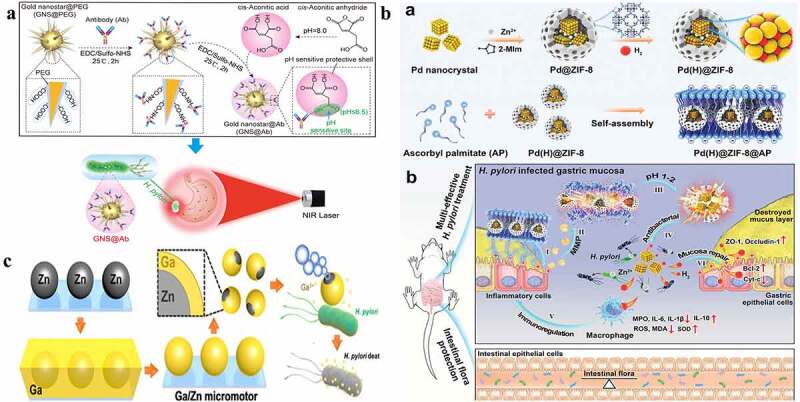
**a)** Schematic preparation of pH-sensitive GNS@Ab and their application in targeted imaging and photothermal therapy of *H. pylori* in vivo with antibiotic resistance; **b)** (a) Schematic diagram of Pd(h)@ZIF-8 and Pd(H)@ZIF-8@AP synthesis and (b) the targeting (i), degradation (II, III), sterilization (IV), regulation of immunity (v), and restoration of gastric mucosa (VI) in the stomach of *H. pylori*-infected mice, and the influence of intestinal flora homeostasis; **c)** Schematic illustration of the fabrication and antibacterial process of Janus Ga/Zn micromotors. **(a) Reproduced with permission from ref**,^25^ **© 2019 Elsevier Inc. (b) Reproduced with permission from ref**,^23^ **© 2021 Wiley‐VCH GmbH**. (c) **Reproduced with permission from ref**,^115^ **© 2021 Wiley‐VCH GmbH.**

Gold nanoparticles are also commonly used inorganic nanoparticles and have also been used for *H. pylori* eradication.^25,112–114^ Gold nanoparticles can deposit on the surface of *H. pylori*, which kills *H. pylori* by membrane disruption. In addition, gold nanoparticles can also enter the cell and produce ROS, which kill *H. pylori* by affecting its metabolism.^25^ For example, Zhi et al. synthesized pH-sensitive acid-sensitive cis-aconitic anhydride-modified anti-*H. pylori*-conjugated gold nanostars (GNS@Ab).^25^ Notably, they improved the bactericidal effect using near-infrared laser photothermal treatment, which reduced the emergence of *H. pylori* resistance and even eradicated *H. pylori*-resistant strains isolated from clinical patients (Figure 5a).^25^ Additionally, most of the GNS@Abs are eliminated from the body after the complete eradication of *H. pylori* in vivo without disrupting the gut-microbiota balance.^25^

Zn and zinc oxide nanoparticles have also been used in anti-*H. pylori* regimens because of their ability to inhibit urease activity and good biocompatibility.^23,116,117^ For example, Zhang et al. integrated Pd(H)@ZIF nanoparticles with ascorbate palmitate (AP, with a negative charge and enzymatic hydrolysis properties) (Figure 5b).^23^ AP can precisely locate *H. pylori*, palladium (Pd) nanoparticles can absorb and release hydrogen (which changes the permeability of the cell membrane and interferes with *H. pylori* metabolism), and the zinc-based zeolitic-imidazolate framework (ZIF-8) can generate Zn^2+^ (which destroys the survival environment of *H. pylori* by inhibiting urease activity).^23^ Notably, Pd(H)@ZIF-8@AP not only kills *H. pylori* but also inhibits the inflammatory response and repairs damaged mucosa by inhibiting the secretion of inflammatory factors in macrophages and removing ROS. In addition, the effect of Pd(H)@ZIF-8@AP on intestinal flora is minimal.^23^ The hydrogen-generating ability of Zn-based micromotors in an acidic environment has been used to design mobile antibacterial platforms. For example, Lin et al. have integrated Zn-based micromotors with gallium ions. The gallium ions present a good ability to eradicate *H. pylori*, and the Ga/Zn micromotors exhibit good locomotivity in a simulated gastric acid environment (pH = 1.5). Moreover, Zn and Ga can degrade completely after 15 min (Figure 5c).^115^

In summary, anti-*H. pylori* mechanisms of inorganic nanoparticles mainly involve oxidative stress, nonoxidative stress, and metal-ion release.^32,112^ Inorganic nanomaterials have gradually gained importance in the field of anti-*H. pylori*, particularly for eradicating drug-resistant bacteria, due to their anti-multidrug resistance and good antibacterial effects. They can effectively eradicate *H. pylori* by multiple antibacterial mechanisms, making it difficult for *H. pylori* to develop resistance, and they can be integrated with other bioactive materials to form multifunctional anti-*H. pylori* platforms. However, their refractory degradation and potential nanotoxicity require further investigation before use in clinical *H. pylori* therapy.

#### Microspheres

Microspheres are homogeneous monolithic particles ranging from 0.1–1000 µm in size that are widely used as controlled-release drug carriers.^118^ Compared with nanoparticles, microspheres exhibit better loading capacities and controlled-release capabilities, facilitating the reduction of drug doses and long-term maintenance of therapeutic effects.^119,120^ Microspheres are often combined with other materials to form multifunctional anti-*H. pylori* platforms. For example, Tripathi et al. designed amoxicillin-loaded pectin/gellan gum-blended sodium alginate microspheres. The results showed that these delivery systems exhibit targeted drug delivery to gastric sites for the treatment of *H. pylori*, along with a suitable drug-release pattern.^121^ Chitosan exhibits excellent adhesion and antibacterial properties,^122^ and chitosan microspheres impart a good loading capacity and controlled-release capability to delivery systems, along with specific adhesion to *H. pylori*.^123,124^ Gonçalves et al. reported that chitosan microspheres exhibit the highest adherence to *H. pylori* at pH 6, and the microspheres are significantly more effective against BabA/SabA^+^*H. pylori* (76% reduction) strain than the BabA^+^/SabA *H. pylori* strain (50% reduction).^123,125^ Moreover, their results showed that chitosan microspheres can eradicate *H. pylori* through adhesion and are subsequently eliminated from the gastrointestinal tract.^126^ Based on this idea, Adebisi et al. also designed a clarithromycin-loaded chitosan hybrid microsphere and found that chitosan causes a significantly high adhesion of the microsphere to *H. pylori*.^124^

Collectively, microspheres exhibit high loading capacities and can sustain drug release over time, increasing the anti-*H. pylori* drug-delivery efficiency while maintaining the bactericidal concentration. In addition, combining with other materials makes microspheres a strong multifunctional anti-*H. pylori* platforms.

#### Hydrogels

Hydrogels are hydrophilic three-dimensional network-structured gels formed by water-soluble or hydrophilic polymers through chemical or physical cross-linking.^127^ As another efficient delivery system for *H. pylori* therapeutic agents, hydrogels exhibit good biocompatibility, degradability, and controlled drug-release capacity.^128–130^ Chitosan and its derivatives are often used as base materials to prepare hydrogels because of their antibacterial and adhesion properties with *H. pylori*.^129,131,132^ For example, El-Mahrouk et al. reported metronidazole-loaded pH-sensitive chitosan hydrogels for *H. pylori* eradication therapy.^131^ The results showed that the swelling and drug-release ability of the hydrogels were highly dependent on pH; they were significant at pH 1.2 but low in phosphate buffers at pH 7.4. In vivo experiments indicated that the system can exist in a dog’s stomach for at least 48 h, and its ability to kill *H. pylori* is stronger than that of oral metronidazole alone.^131^ Similarly, Mohamed et al. have developed a novel chitosan hydrogel. Notably, they linked the chitosan hydrogel with benzophenone tetracarboxylimide benzoyl thiourea, which selectively inhibited cyclooxygenase-2 activity.^129^ This means that hydrogels can also function as multifaceted platforms for *H. pylori* eradication. Additionally, hydrogels also exhibit high drug-loading capacity and the ability to load multiple anti-*H. pylori* agents.^133^ For example, Silva et al. fabricated cross-linked sodium alginate-carboxymethyl cellulose hydrogels loaded with furazolidone (encapsulation efficiency: 71–76%) and bismuth(III) (encapsulation efficiency: 88%).^133^ The hydrogels swelled to approximately 150% in the first 2 h, remaining for at least 6 days in simulated gastric tissue (pH = 1.2), indicating that the hydrogels can exhibit sustained drug release. Although this study did not show the role of hydrogels in eradicating *H. pylori*, it reported their drug-loading capacity and therapeutic potential toward *H. pylori*.

Hydrogels, with high drug-carrying capacity, are efficient anti-*H. pylori* drug-delivery systems. They exhibit numerous advantageous properties; their three-dimensional network structure improves drug stability, and they exhibit good biocompatibility and sustained drug-release ability.^134^ Targeting the low drug utilization rate of anti-*H. pylori* drugs, hydrogels can effectively improve medication utilization by maintaining a sustained release of drugs in the stomach, effectively eradicating *H. pylori* and hindering the development of drug resistance.^135^

## Conclusion and future perspective

**Figure 6.**
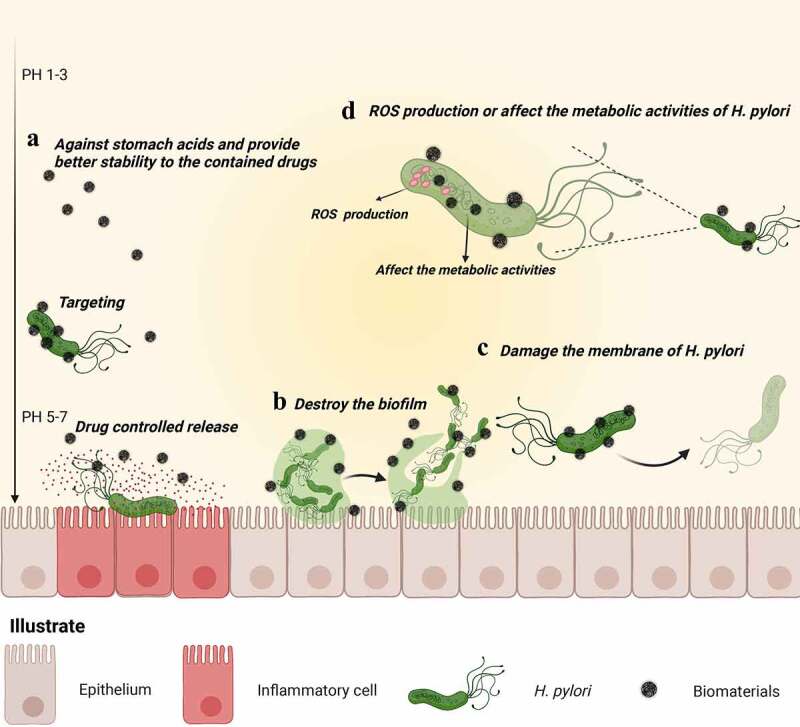
**Schematic representation of the main mechanism of *H. pylori* eradication by biomaterials**. (a) Acting as a physical barrier against stomach acids and enzymes, biomaterials provide better stability to the contained drugs. Furthermore, biomaterials delay drug release and target lesions, increasing the contact between *H. pylori* and the drug, thereby improving drug utilization. (b) Biomaterials directly destroy biofilms (one of the causative factors for *H. pylori* antibiotic resistance), eradicating *H. pylori* from biofilms. (c) Biomaterials damaging *H. pylori* membranes. (d) Biomaterials (such as inorganic metal materials) themselves exhibit antibacterial effects that kill *H. pylori* directly.

The increasing prevalence of antibiotic-resistant strains and high failure rate of treatment are severe challenges to the current *H. pylori* treatment strategies. Thus, extensive research has been carried out to find effective strategies to combat *H. pylori* infections and antibiotic resistance, including designing new drug combinations, extending the treatment periods, and performing susceptibility testing before treatment. However, the existence of gastric acid milieud and pepsin, which can destroy most of the antibiotics in the stomach, and gastric mucosa, which is approximately 200 μm thick with a pH gradient from pH 1–2 to 7,^32^ cripple effective antibiotic delivery efficiency. This makes drug eradication programs extremely complicated, which not only significantly affects patient compliance but also increases eradication costs and consequently limits the applicability of H. pylori therapy. Therefore, improving drug delivery efficiencies and developing effective antibiotic-independent antimicrobial moieties could be promising emerging approaches to resolve the challenges associated with anti-*H. pylori* processes. As a new strategy to eradicate *H. pylori*, biomaterials have the following advantages (Figure 6): (1) Protected drugs against stomach acid, targeted lesions and controlled drug release. With increasing antimicrobial resistance, the optimization of dosing and delivery efficiency can produce higher eradication rates. The biomaterials summarized in this review, such as nanoparticles, microspheres, and hydrogels, can protect drugs against the harsh acidic environment of the stomach and exhibit controlled drug-release capacity with high delivery efficiency. Among them, microspheres and hydrogels exhibit particularly high drug loading and controlled drug-release capacities and can deliver multiple anti-*H. pylori* drugs.^136,137^ Therefore, if we want to deliver multiple anti-*H. pylori* drugs, microspheres and hydrogels may be a good choice. For example, it may be interesting to see the eradication effects of vonoprazan dual therapy (a novel eradication regimen for *H. pylori*) delivered by microspheres or hydrogels in the future. Targeted lesions are another strategy for improving delivery efficiency, thereby increasing eradication rates and reducing the development of *H. pylori* resistance. Biomaterials (such as chitosan and ascorbate palmitate) can adhere to *H. pylori* through electrostatic interactions, targeting lesions, and indirectly prolonging the duration of drug action. However, Gottesmann et al. indicated that electrostatic interactions alone are not sufficient for specific interactions with *H. pylori*, and direct interactions with the *H. pylori* surface (such as specific binding with the mucin of *H. pylori*) are critical.^76^ Hence, this should be considered when developing bacteria-targeting biomaterials. (2) Destroys the biofilm. Biofilms play an important role in the emergence of *H. pylori* antibiotic resistance, and the antimicrobial resistance of bacteria growing inside biofilms can be 1000 times higher than that of external bacteria.^100^ Biomaterials, such as rhamnolipids and silver, can bind to the extracellular substance of biofilms and disrupt them by mediating signals or by other means. Therefore, strategies involving these biomaterials could be powerful “weapons” against the antibiotic-resistance crisis. (3) Changes in the permeability of the *H. pylori* membrane. Biomaterials such as linolenic acid, docosahexaenoic acid, and nanostructured lipid carriers can change the permeability of the *H. pylori* membrane, disrupting its integrity. Additionally, owing to the presence of components similar to those of cell membranes, these biomaterials could also have sterilizing effects on intracellular *H. pylori* compared with other biomaterials. Hence, these biomaterials could be used for the development of antibiotic-free anti-*H. pylori* agents. In addition, chitosan can also change the permeability of the *H. pylori* membrane; unlike the aforementioned biomaterials, its mechanism could involve its adhesion to *H. pylori*, followed by membrane destruction by the physiochemical properties of the nanomaterial. (4) Produced reactive oxygen species (ROS) or affected the metabolic activities of *H. pylori*. Apart from mechanically disrupting its membrane through the direct contact of nanomaterials with *H. pylori*, some biomaterials (such as gold nanoparticles, liquid metal gallium, Zn, and zinc oxide nanoparticles) eradicate *H. pylori* by generating ROS or by disrupting its metabolic activities. A large amount of ROS causes changes in bacterial morphology and DNA damage. Owing to their unique anti-*H. pylori* mechanisms, these biomaterials could also be suitable for developing antibiotic-free anti-*H. pylori* agents (Table 2).

**Table 2.** The current anti-*H. pylori* mechanism of biomaterials.

Anti-H. Pylori mechanisms	Biomaterials	Examples	Reference
Protect drugs against stomach acid	Liposomes	Liposomal linolenic acid system, Liposomes with encapsulated furazolidone	^24,74^
	Nanoemulsion	Nanoemulsion-based delivery systems loaded with erythromycin	^87^
	Nanostructured lipid carriers	DHA-loaded nanostructured lipid carriers	^89^
	Polymeric nanoparticles	Chitosan nanoparticles, PH-responsive chitosan-heparin nanoparticles	^94,97^
	Inorganic nanoparticles	GNS@Ab	^25^
	Microspheres	Chitosan microspheres, Clarithromycin-loaded chitosan hybrid microsphere	^123,124^
	Hydrogel	PH-sensitive chitosan hydrogels loaded with metronidazole	^131^
Target lesions	Liposomes	Pectin-coated liposomes with encapsulated amoxicillin	^76^
	Polymeric nanoparticles	Amoxicillin-loaded genipin-cross-linked fucose-conjugated chitosan/heparin nanoparticles	^96^
	Inorganic nanoparticles	Pd(H)@ZIF nanoparticle with ascorbate palmitate	^23^
Controlled drug release	Liposomes	Liposomes-polymer hybrid nanoparticles loaded with amoxicillin	^78^
	Nanoemulsion	Nanoemulsion-based delivery systems loaded with erythromycin	^87^
	Nanostructured lipid carriers	DHA-loaded nanostructured lipid carriers	^89^
	Polymeric nanoparticles	Superparamagnetic iron oxide nanoparticles coloaded with amoxicillin and chitosan/polyacrylic acid particles	^95^
	Microspheres	Chitosan microspheres, Clarithromycin-loaded chitosan hybrid microsphere	^123,124^
	Hydrogel	Benzophenone tetracarboxylimide benzoyl thiourea linked chitosan hydrogel	^129^
Destroy the biofilm	Liposomes	Liposomes-polymer hybrid nanoparticles loaded with amoxicillin	^78^
	Polymeric nanoparticles	Rhamnolipids-chitosan hybrids nanoparticles loaded clarithromycin	^98^
	Inorganic nanoparticles	Ag(PhTSC∙HCl)2] (NO3) ∙H2O polymeric nanoparticles	^111^
Change membrane’s permeability	Liposomes	Liposomal linolenic acid system	^24^
	Nanostructured lipid carriers	DHA-loaded nanostructured lipid carriers	^89^
	Polymeric nanoparticles	Chitosan nanoparticles, PH-responsive chitosan-heparin nanoparticles	^94,97^
	Inorganic nanoparticles	Pd(H)@ZIF nanoparticle with ascorbate palmitate	^23^
ROS production	Inorganic nanoparticles	GNS@Ab, Ga/Zn micromotors	^25,115^

From bench to bedside, numerous challenges need to be addressed before designing biomaterials for the efficient treatment of *H. pylori* infections in the future. First, the designed biomaterials should exhibit high stability in the harsh acidic environment of the stomach to improve the bioavailability of drugs. Second, to ensure good anti-*H. pylori* activity by biomaterials, bacterial targeting should be applied in the formulation design. Third, rapidly increasing *H. pylori* resistance is largely attributable to the misuse of antibiotics. Delivering antibiotics with low resistance rates (such as amoxicillin), improving delivery efficiency, and developing antibiotic-independent antimicrobial moieties could be the focus of future studies. Fourth, the persistent survival of *H. pylori* inside cells and biofilms is also an important reason for therapeutic failure and the emergence of *H. pylori* resistance. Therefore, fabricating biomaterials that can eradicate *H. pylori* present intracellularly and inside biofilms is crucial for improving the cure rate. Additionally, it is also vital to maintain the ecological homeostasis of the gut ecosystem and develop biodegradable biomaterials for *H. pylori* treatment. Most importantly, clinical translation and widespread applications are the ultimate goals of biomaterial design, making cost effectiveness and large-scale production important considerations.

In summary, biomaterials can be used to encapsulate and deliver conventional antibiotics, antimicrobial lipids, vaccines, or phytomedicines with antibacterial effects to increase the drug delivery efficiency and hinder the development of drug resistance in *H. pylori*. Their unique multiple antimicrobial mechanisms eradicate *H. pylori* that is present intracellularly and inside biofilms and could solve the current problem of high drug resistance. This review discusses recent progress in biomaterials for the eradication of *H. pylori* and summarizes the main anti-*H. pylori* mechanisms. In conclusion, biomaterials exhibit high potential to be integrated into standard treatments in the future because of their safety and *H. pylori* eradication efficiency. The application of biomaterials could help achieve the ultimate goal of complete elimination of *H. pylori* in the future.
